# Exploring diabetic foot screening programs with integrated consolidated framework for implementation: Rapid review protocol

**DOI:** 10.12688/hrbopenres.14119.1

**Published:** 2025-04-04

**Authors:** Virginie Blanchette, Maya Fakhfakh, Yassin Andoulsi, Magali Brousseau-Foley, Jennifer A Pallin, Claire Buckley, Laura M Drudi, Charles de Mestral, Janet L Kuhnke, Caroline McIntosh

**Affiliations:** 1 Department of Human Kinetics and Podiatric Medicine, University of Quebec at Trois-Rivieres, Trois-Rivières, Québec, G8Z 4M3, Canada; 2 VITAM Center for Sustainable Health Research, Québec City, Québec, G1J 2G1, Canada; 3 Faculty of Medicine,, University Laval, Québec, Québec, G1V 0A6, Canada; 4 School of Population Health, Royal College of Surgeons of Ireland, Dubblin, D02 YN77, Ireland; 5 School of Public Health, University of Cork, Cork, Cork county, Ireland; 6 University of Montreal Hospital Centre, Montreal, Québec, Canada; 7 Department of surgery, University of Toronto, Toronto, Ontario, Canada; 8 Division of Vascular Surgery, Saint Michael Hospital, Toronto, Canada; 9 School of Nursing,, Cape Breton University, Sydney, Nova Scotia, Canada; 10 Discipline of Podiatric Medicine, School of Health Sciences, University of Galway, Galway, County Galway, Ireland

**Keywords:** Foot ulcer; Diabetic Foot; Prevention and Control; Implementation Science; Primary Health Care; Diagnostic Screening Programs

## Abstract

**Background:**

Diabetic foot ulcers (DFU)s pose significant challenges for individuals with diabetes, leading to severe consequences, such as lower extremity amputations (LEA)s, reduced quality of life, and increased mortality. Disorganized diabetic foot care services contribute to health inequities worldwide, highlighting the need for structured preventive measures, which require an understanding of organizational and systemic components of the implementation of foot screening programs or initiatives, including equity factors. Thus, the *Consolidated Framework for Implementation Research* (CFIR) is one of the most widely used frameworks for assessing these factors and contexts. This helps to reduce the risk of failure of implementation efforts in the real world and can help to support the scaling up of preventative measures. This review aims to analyze foot screening programs or initiatives for individuals at risk of DFUs and LEAs, define their key components and implementation determinants, identify barriers and facilitators, and describe effective implementation strategies in primary care with CFIR.

**Methods:**

A rapid review will be conducted following the Canadian method by Dobbins (2017) and reported according to the Preferred Reporting Items for Systematic Reviews and Meta-Analyses Protocol guidelines. The research question is defined using the PICO framework. A systematic search will be conducted in MEDLINE, CINAHL, and EMBASE. Primary studies in English or French, including both primary study designs and knowledge syntheses, will be screened according to the defined eligibility criteria via Covidence. Study quality will be appraised using the Mixed Methods Appraisal Tool and data will be synthesized guided by the CFIR. Data synthesis will focus on implementation determinants, including barriers, facilitators, and implementation strategies.

**Discussion:**

Findings will inform policy, practice and decision making regarding the implementation of screening programs. This can promote the development of screening programs for diabetic foot complications across Canada or in other countries.

## Introduction

Foot health is a significant public health concern for individuals living with diabetes ^1^. Diabetic foot disease (DFD) is one of the most severe complications of diabetes, affecting 199 million people globally, and accounting for 2% of the total disease burden, mortality associated with diabetic foot ulcers (DFU) may be as high as 50% in 5-year ^2^. DFU can give rise to severe consequences for affected individuals, including lower extremity amputations (LEA) ^3, 4^, reduced quality of life ^5, 6^, premature death ^7, 8^, and contribute to substantial direct and indirect costs for both individuals and the healthcare system ^9, 10^. This underscores the significant burden of diabetic foot diseases on the healthcare system and individuals. Studies also show that individuals with diabetes fear LEAs more than death ^11^, and often feel inadequately supported by professionals on medical, educational or emotional levels to care for their feet ^12^. Up to 85% of amputations are preceded by a preventable foot ulcer yet a limb is lost to diabetes every 20 seconds across the globe ^13^. Currently, treatments and research largely focus on addressing advanced foot disease rather than prevention ^14^. Shifting to prevention is crucial, as evidenced by successful approaches in other diseases like cancer, where screening and awareness campaigns for early detection, such as mammograms and human papilloma virus (HPV) vaccinations, have significantly reduced mortality, treatment costs and improved patient quality of life ^15^. A similar preventive approach in diabetic foot care, including regular foot screenings, patient education, and lifestyle interventions, is warranted. However, in reality, this is not happening.

Preventing LEA needs a specialized and dedicated team approach ^16^. Moreover, preventing DFUs involves integrated healthcare in primary care to specialized care, including screening for foot complications—a specific strategy to improve diabetes care quality in primary care ^17^. However, care and services for diabetes-related foot care are disorganized in Canada in other countries, contributing to health inequities ^18^. Hence, structural actions are required to address these disparities, especially with populations in vulnerable contexts, such as Indigenous Peoples populations in rural or remote communities or disadvantaged populations, who are notably facing exacerbated consequences ^19– 21^. One of the ways to better structure healthcare services when it comes to DFU and LEA prevention is putting in place foot screening initiatives or programs, especially in primary and community care as suggested by the 2023 International Working Group on Diabetic Foot (IWGDF) ^2^. This approach enables knowledge mobilization in all healthcare, ensuring healthcare service delivery to individuals with diabetes at the right time ^22, 23^. However, although foot screening involves simple, acceptable, and cost-effective actions at the individual level, its population-based implementation is more complex at the organizational and societal levels, considering various factors, such as the rising number of individuals with diabetes, local contexts, resource availability, intervention, and organizational heterogeneity ^2, 24, 25^.

According to the IWGDF, implementing screening for individuals at risk of DFUs is a prioritized research area, with efforts to focus on limb preservation programs for high-risk individuals ^2^. This is also a strategic direction outlined in the Canadian Diabetes Framework ^26^. Moreover, avoiding LEAs and DFUs with primary prevention is a priority identified by the patient partners involved in this patient-oriented research as well as by many individuals with diabetic foot disease in the literature ^12^. Thus, the scientific literature on this topic can serve as a crucial source of data regarding implementation, supporting knowledge applications facilitating the translation of knowledge into action ^27^. However, to our knowledge, none of the previous knowledge synthesis related to this topic ^24, 25, 28, 29^ has explored the existing landscape of screening programs for diabetic foot disease, especially in primary care, integrating the Consolidated Framework for Implementation Research (CIFR) ^30^. CFIR is one of the most widely used and cited frameworks for evaluating implementation factors and contexts and can help to reduce the risk of failure of implementation efforts in the real world ^30^. CFIR provides an in-depth understanding of what influences implementation outcomes, and although it did not initially focus on equity, it has been adapted to include related sub-constructs ^30^. It can also help to support scaling ^31^. CFIR proved useful and comprehensive in evaluating and identifying the contextual factors at several levels that have an impact on the implementation of diabetes initiatives by identifying the gaps between ideal care and reality, evidence-based solutions to address gaps and barriers to implementing solutions ^32– 34^. Recently, Houghton *et al.* (2025) explored the barriers and the facilitators of evidence-based foot screening with an implementation sciences lens ^35^. This was an important step in supporting the implementation of research in real-life practice. As implementation science is a recent field of research ^36^, analyzing the literature specifically with CFIR will further explore the challenges associated with implementing diabetic foot screening initiatives/programs and discern effective implementation strategies to optimize prevention, as there is currently no consensus specifically dedicated to DFS screening in Canada ^29^.

## Objectives

Given the recent knowledge syntheses on screening for diabetic foot disease ^24, 25, 29, 35^ and the increased interest in the impacts of this intervention, the aim of this project is to conduct a secondary analysis of the literature, identified with a rapid review, of foot screening initiatives or programs using the principles of implementation mapping ^30, 37, 38^. This will make it possible to identify various data from the scientific literature from the CFIR's point of view to possibly enable the development of an optimized screening program for primary care and the real context.

The specific objectives are:

1) To define the components of diabetic foot screening initiatives/programs from a supplemented Consolidated Framework of Implementation Research (CFIR) perspective, including the equity lens ^39^;

2) To describe the barriers and facilitators inherent in the implementation of foot screening initiatives/programs, particularly in the primary care setting for individuals at risk of DFU and/or LEA;

3) To describe implementation strategies to support foot screening initiatives/programs, particularly in primary care for individuals at risk of DFU and/or LEA using the “Expert Recommendations for Implementing Change (ERIC) tool ^40, 41^.

## Methods

### Study design

A rapid review will be conducted, based on an adapted 5-step process from the Canadian method suggested by Dobbins (2017) ^42^. Yet, there is no method proven optimal related to this research design ^43, 44^. This research design was preferred given that it is a subject of growing interest and that the relevant scientific literature can serve as a crucial source of data concerning implementation and facilitate the translation of knowledge into action ^27^, thus accelerating the search for answers that will affect the development and implementation of an appropriate screening program in Canada and providing rapid information on what can influence organizational and political decisions regarding this health issue.

The rapid review will be reported using the recommendations by the Cochrane Rapid Review Methods Group ^45^ and will follow an adapted version of the PRISMA-P reporting guidelines (Appendix 1) ^46^. The rapid review will be registered with Open Science Framework ( osf.io/ghyxz).

***Step 1: Define the question, the concepts and eligibility criteria***

Following the PICO framework, the research question is related to the specific objectives of this rapid review ^47^: What is the scientific evidence for mapping the components of the diabetic foot screening initiative/program from an implementation perspective for the population at risk of DFU and/or LEA?

•  Population (P): Individuals with diabetic foot disease (type 1 or 2 diabetes; aged >18 years), at-risk of DFUs and/or LEAs, as defined by the IWGDF ^48^.

•  Intervention (I): Comprehensive prevention initiative/programs for foot screening in individuals at-risk of DFUs and LEAs. A foot screening initiative is defined as an effort by a health care professional, a health care team, an organization, or a health care system for population-based foot screening. A foot screening program is defined as structured and integrated intervention(s) that can use any of the risk stratification tools and any assessment of the vascular (e.g., arterial blood supply, with the use of ankle-brachial pressure index or structured clinical examination of peripheral pulses), the dermatology (e.g., nails and callus, previous LEA and/LEA), the neurology (e.g., loss of protective sensation), and foot deformities (e.g., hammer toes, Charcot Foot, including shoes evaluation), that are supported by the IWGDF ^49^, in a cohort of individuals with diabetes. The initiative or program may be a component of broader activities for diabetes prevention/management.

•  Comparator (C): No restrictions, i.e., none, or standard intervention as the control group dependent on the study design.

•  Outcomes (O): Implementation determinants as described by the Consolidated Framework for Implementation Research (CIFR) and adapted to the present study, including barriers and facilitators, implementation strategies and outcomes ^30, 37, 38^. These are presented in Table 1 and Table 2 (Extended data)

**Table 1.** Implementation Determinants from CFIR Domain relabeled for foot screening initiatives/programs, adapted from *Damschroder et al. 2009* and *Grant et al., 2024* ^52, 53^. https://cfirguide.org/.

CFIR Domain	Relabeled	CFIR Constructs ^†^	CFIR Sub-constructs	Description
I. Intervention Characteristics	Features of the foot screening initiative/program Implementation and Effectiveness.	8	0	This domain contains 8 constructs related to beliefs, perceptions, and characteristics of the intervention (single or complex interventions integrated into the initiative/program), which are defined as the implementation or creation of the foot screening initiative/program.
II. Outer Setting	Government, Health Authorities and Health Organizations.	7	3	This domain is defined as the collaborative and/or integrated foot screening initiative/program and includes 7 constructs.
III. Inner Setting	Characteristics of the foot screening initiative/program.	10	10	This domain refers to the health care practice related to the foot screening initiative/practice (i.e., the entity of a practice, which includes the health professionals, administration, manager setting, etc.), may be multi-levels (e.g., hospital, team) and consists of 10 constructs.
IV. Characteristics of Individuals	Characteristics of all the Individuals integrated in the foot screening initiative/program.	Roles (4) Characteristics (4)	0	This domain refers to any individuals working within the initiative/program and includes 8 constructs.
V. Process	Features of the Process of Implementation of the foot screening initiative/program.	9	6	This domain refers to the implementation of the foot screening initiative/program, including barriers, facilitators, strategies and knowledge mobilization initiatives, and includes 9 constructs.

† Description of the constructs: https://cfirguide.org/

**Table 2.** Constructs from CFIR Domain relabeled for foot screening initiative/program, adapted from *Damschroder et al. 2009* ^52^.

CFIR Domains ^†^	Adapted CFIR Constructs ^†^	Adapted Definitions
I. Intervention Characteristics	A. Foot Screening Initiative/Program Source B. Foot Screening Initiative/Program Evidence-Base C. Foot Screening Initiative/Program Relative Advantage D. Foot Screening Initiative/Program Adaptability E. Foot Screening Initiative/Program Trialability F. Foot Screening Initiative/Program Complexity G. Foot Screening Initiative/Program Design H. Foot Screening Initiative/Program Cost	A. The group/organization/team/researcher that developed and/or visibly sponsored use of the initiative/program is reputable, credible, and/or trustable. B. The initiative/program has robust evidence supporting its effectiveness and integrated IWGDF guidelines and best practice recommendations for all included interventions. C. The initiative/program is better than other available initiative/program or current practices (e.g., comparator of the study). D. The initiative/program can be modified, tailored, or refined to fit local context or needs (e.g., flexibility) E. The initiative/program can be/has been tested or piloted on a small scale and undone (e.g., seek out to preliminary results/pilot). F. The initiative/program is complicated, which may be reflected by its scope and/or the nature and number of connections and steps (e.g., trajectories, many stakeholders, timeframes, etc.). G. The initiative/program is well designed and packaged, including how it is assembled, bundled, and presented (e.g., reproducible of the research project vs. reporting guidelines of the research design) H. The initiative/program purchase and operating costs are affordable (exposed) (e.g. Technologies, basic materials, human resources needed to run the program, etc.).
II. Outer Setting	A. Critical Incidents B. Local Attitudes beliefs, norms C. Local Conditions D. Partnership & Connections E. Policy & Laws F. Financing G. External Pressures	A. Large-scale and/or unanticipated events disrupt implementation and/or delivery of the initiative/program (e.g., pandemic conditions [e.g., Covid], Adverse event). B. Sociocultural values (e.g., shared responsibility in helping individuals with diabetes) and beliefs (e.g., convictions about the worthiness of individuals with diabetes in the initiative/program) encourage the Outer Setting to support the implementation and/or delivery of the initiative/program. C. Economic, environmental, political, and/or technological conditions enable the Outer Setting to support the implementation and/or delivery of the initiative/program. D. The Inner Setting is networked with external entities, including referral networks, academic affiliations, and professional organization networks. E. Legislation, regulations, professional group guidelines and recommendations, or accreditation standards support the implementation and/or delivery of the initiative/program. F. Funding from external entities (e.g., grants, reimbursement) is available to implement and/or deliver the initiative/program. G. External pressures drive implementation and/or delivery of the initiative/program. See subconstructs related to Societal, Market or Performance-measurement pressure.
III. Inner Setting	A. Structural characteristics (physical infrastructure; information technology infrastructure, work infrastructure) B. Relational Connections and Communication C. Culture (Human Equality-Centredness, Individual with diabetes-Centredness, Deliver-Centredness, Learning-Centredness) D. Tension of Charge (specific to the implementation and/or delivery of the foot screening initiative program) E. Compatibility F. Relative Priority G. Incentive Systems H. Mission Alignment I. Available Resources (funding, space, material & equipment) J. Access to knowledge & Information	A. Infrastructure components support functional performance of the Inner Setting. Physical Space: Layout and configuration of space and other tangible material features support functional performance of the Inner Setting. Information technology: Technological systems for telecommunication, electronic documentation, and data storage, management, reporting, and analysis support functional performance of the Inner Setting. Work: Organization of tasks and responsibilities within and between individuals and teams, and general staffing levels, supports functional performance of the Inner Setting. B. There are high quality formal and informal information sharing practices within and across Inner Setting boundaries (e.g., structural, professionals). C. There are shared values, beliefs, and norms across the Inner Setting, about the inherent equal worth and value of all human beings, around caring, supporting, and addressing the needs and welfare of individuals with diabetes and the delivers, around psychological safety, continual improvement, and using data to inform practice. D. The current situation is intolerable and needs to change. E. The foot screening initiative/program fits with workflows, systems, and processes. F. Implementing and delivering the foot screening initiative/program is important compared to other initiatives. G. Tangible and/or intangible incentives and rewards and/or disincentives and punishments support the implementation and delivery of the foot screening initiative/program. H. Implementing and delivering the foot screening initiative/program is in line with the overarching commitment, purpose, or goals in the Inner Setting. I. Resources are available to implement and deliver the innovation. Funding is available to implement and deliver the foot screening initiative/program. Physical space is available to implement and deliver the foot screening initiative/program. Supplies are available to implement and deliver the foot screening initiative/program. Communication of results to the greater interprofessional team J. Guidance and/or training is accessible to implement and deliver the foot screening initiative/program.
IV. Characteristics of Individuals	Roles A. Types of leaders (i.e., high-level, mid-level, Opinions, Implementation Facilitators, Implementation Lead, Implementation team members) B. Other Team Support C. Diabetic foot screening initiative/program Delivers D. Diabetic foot screening initiative/program Individuals with Diabetes Characteristics E. Need F. Capability G. Opportunity H. Motivation	A. Individuals with a high level of authority, including key decision-makers, executive leaders, or directors. Individuals with a moderate level of authority, including leaders supervised by a high-level leader and who supervise others. Individuals with informal influence on the attitudes and behaviours of others. Individuals with subject matter expertise who assist, coach, or support implementation. Individuals who lead efforts to implement the diabetic foot screening initiative/program. Individuals who collaborate with and support the Implementation Leads to implement the diabetic foot screening initiative/program, ideally including the diabetic foot screening initiative/program deliverers and individuals with diabetes. B. Individuals who support the Implementation Leads and/or Implementation Team Members to implement the diabetic foot screening initiative/program. C. Individuals who are directly or indirectly delivering the diabetic foot screening initiative/program. D. Individuals who are directly or indirectly receiving the diabetic foot screening initiative/program. E. The individual(s) has deficits related to survival, well-being, or personal fulfillment, which will be addressed by the implementation and/or delivery of the diabetic foot screening initiative/program. F. The individual(s) has interpersonal competence, knowledge, and skills to fulfill roles (e.g., accreditation, professional competencies) G. The individual(s) has availability, scope, and power to fulfill roles (e.g., inspiration, why). H. The individual(s) is committed to fulfilling Roles (e.g., motivations being the initiative/program).
V. Process	A. Teaming B. Assessing Needs (Delivers and Individuals with Diabetes Recipients) C. Assessing the context D. Planning E. Tailoring Strategies F. Engaging (Delivers and Individuals with Diabetes) G. Doing H. Reflecting and Evaluating (Implementation and Foot Screening Initiative/Program) I. Adapting	A. Join together, intentionally coordinating and collaborating on interdependent tasks, to implement the foot screening initiative/program. B. Collect information about the priorities, preferences, satisfaction and needs of the Delivers Individuals with diabetes to guide the implementation and delivery of the foot screening initiative/program. C. Collect information to identify and appraise barriers and facilitators to the implementation and delivery of the foot screening initiative/program. D. Identify roles and responsibilities, outline specific steps and milestones, and define goals and measures for implementation success in advance of the foot screening initiative/program (See implementation outcomes). E. Choose and operationalize implementation strategies to address barriers, leverage facilitators, and fit context. F. Attract and encourage participation of delivers and Individuals with diabetes in the implementation and/or the foot screening initiative/program. G. Implement in small steps, tests, or cycles of change to trial and cumulatively optimize delivery of the foot screening initiative/program. H. Collect and discuss quantitative and qualitative information about the success of the implementation. I. Modify the foot screening initiative/program. and/or the Inner Setting for optimal fit and integration into work processes.

† Description of the Domains and Constructs: https://cfirguide.org/ Abbreviations: IWGDF: International working group on diabetic foot

Implementation Outcomes ^50^:

- Acceptability
- Adoption
- Appropriateness
- Cost
- Feasibility
- Fidelity
- Penetration
- Sustainability
- Benefits/harms
- Quality
- Performance

Intervention/System Outcomes

- Efficiency/Effectiveness (e.g., LEAs major/minor, 1st DFU prevention, hospitalization, mortality, etc.)
- Safety
- Equity
- Patient-centredness
- Timeliness
- Reliability
- Ethics
- Implementation Strategies

The eligibility criteria (inclusion and exclusion criteria) are defined by the PICO as outlined below:

•  Population: Exclusion of persons/studies high-risk feet that have undergone amputations or have a history of DFUs.

•  Settings: All care settings will be considered. However, we intend to have a particular focus on primary care, as defined by the World Health Organization, these settings support “first contact, accessible, continuous, comprehensive and coordinated person-focus care with the aim of optimizing population health and reducing disparities across populations by ensuring that subgroups have equal access to service” ^51^.

•  Study Design: We will consider: 1) knowledge synthesis (i.e., systematic review, umbrella review, scoping reviews, meta-synthesis, rapid reviews, integrative reviews) with a systematic search method only; 2) primary qualitative, quantitative, or mixed-method study (i.e. observational (case control, cohort, case series, cross-sectional), randomized and non-randomized control trial, qualitative and economic evaluation study designs). Abstracts, guidelines, protocols, expert opinions, editorials and grey literature will be excluded.

•  The time frame of published literature: For knowledge syntheses, we will search the database from inception to date. However, we will only consider primary studies after September 2023, which is the latest systematic search from *Staniszewska et al.* 2024 ^24^, identified by the team as the most recent review on this topic in our preliminary search.

•  Language: We consider all the studies published in French or English.

***Step 2: Search for research evidence***

**Information sources**

A three-step systematic search strategy will be collaboratively developed with an academic librarian using keywords (and their truncate terms) and medical subject heading terms. Boolean operators “AND” and “OR” and proximity operators will combine search terms to ensure our strategy is as efficient as possible and to reduce the risk of capturing irrelevant material. Step 1 aims to identify all knowledge syntheses on the topic of interest, while Step 2 aims to retrieve primary published studies since the publication of the last knowledge synthesis identified. We will compare our search strategies with that of the knowledge syntheses on the subject of interest that the team has identified in our preliminary research ^24, 25, 28, 29^, including studies ^25, 29^ conducted by some of the authors of this project. The overall search strategy will encompass a full range of databases, namely Medline, CINAHL and EMBASE, to ensure coverage of standard sources. Step 3 will involve using keyword combinations and the *Google scholar* search engine and will search the reference lists of all the selected studies to identify any additional relevant studies.

Thus, the source of information for the studies to be screened is all the primary studies included in step 1 and those retrieved in steps 2 and 3. For this reason, this project is a secondary analysis of literature. Initial searches will cover the creation period of each database up to the current date (step 1), while the other (step 2) will be executed with specific date ranges to include the most recent and relevant studies. The entire reference management process will be facilitated by Endnote (Clarivate Analytics, London). An example of a search strategy (Medline) is presented in Appendix 2 and will be adapted according to the database.

**Data selection and screening**

All the studies included in the knowledge syntheses (step 1) will be manually reviewed after their integration into the Endnote database along with those from steps 2 and 3, and then screened according to inclusion and exclusion criteria.

A rapid review does not require a second independent reviewer for the selection phase. Therefore, a single reviewer (MF) will review the title, abstract and full text. However, the second independent reviewer (YA) will review the included studies to certify inclusion criteria. The second reviewer (YA) will support data extraction to ensure a rigorous and efficient review of the selected literature, as secondary analysis may require interpretation of the data. Conflicting decisions between the two independent reviewers (MF and YA) will be resolved through consultation with the principal investigator (VB). A PRISMA flow diagram will be created to ensure transparency of reporting the selection process.

***Step 3: Critically appraise the information source***

The included and excluded studies will be reviewed by the principal investigator (VB) to ensure the accuracy and consistency of the inclusion/exclusion criteria. In addition, the quality of the selected studies will be assessed using the Mixed Methods Appraisal Tool (MMAT) adapted for various study designs ^54^. The risk of bias evaluation will be integrated with the data extraction process, providing a comprehensive analysis of both study content and methodological robustness. This will be performed by the first reviewer (MF).

***Step 4: Extract and synthesize the evidence***

**Data extraction**

At this stage, all the studies to be extracted will be primary studies corresponding to the inclusion criteria. A pilot test of the data extraction form (3 studies) will be carried out by two independent reviewers (MF; YA) and the data extraction process/sheet will be refined by the team if necessary. Data extraction will then be performed by the first reviewer (MF) and verified by a second reviewer (YA). Any discrepancies will be resolved by consensus with the principal investigator (VB). Extracted data will include:

•  Study characteristics (title, authors, years of publication, country, objectives, research designs, including co-design approaches (user-controlled approach, consultative approach, real patient stories, others), study setting; funding);

•  Population (sample size, participant characteristics, including equity-related factors (PROGRESS+ factors and type of diabetes));

•  Foot screening intervention implemented (single or complex intervention and its features ^55^); description of foot screening initiatives/programs, including tests used, education components and resources, risk stratification, pathways/care trajectories, individuals involved in foot screening, such as the role of patients (and care partners), team (who), community, frameworks, guidelines and/or best practice recommendations used, etc.);

•  Possible comparator

•  Foot screening initiative/program outcomes (determinants) from an implementation perspective ( Table 1 and Table 2) ^30, 37, 56^. The extraction will seek to collect specific data on barriers, facilitators (enablers) and factors influencing implementation, as well as strategies used in the studies to overcome challenges and/or suggestions for further work.

•  Alignment with culturally responsive initiatives/programs (e.g., self-knowledge, communication, emphasis on power sharing and dialogue);

•  Knowledge translation activity related to the initiative/program (integrated knowledge translation, knowledge creation, dissemination, implementation, sustainability, scalability and evaluation).

**Data synthesis**

The PRISMA flowchart will provide the synthesis of the selection/screening process. A narrative synthesis approach will be used to report information from the included studies, guided by the CFIR domains (Appendix 3) in five key areas: characteristics of the foot screening initiative/program, including equity factors and complex intervention components if present, the external context, the internal context (organization and infrastructure), individuals (motivation and leadership), and the implementation process (strategies) ^30^. This approach will help to generate a comprehensive list of barriers and facilitators, the associated strategies, and define the recurring components of the initiatives/programs based on the literature. The research team will reflect on the implication of these findings in the implementation of foot screening initiatives/programs to support implementation science on this topic. The overall strength of the body of evidence will not be assessed using the GRADE approach ^57^. Instead, the team will discuss the robustness and quality of the data in relation to the biases of the articles included and the elements present or missing in relation to the CFIR. The GRADE approach was not preferred because this review does not aim to make clinical recommendations, but mainly to identify elements of the process between the core components of foot screening and what influences it in order to adapt to a given environment, including individuals involved, outer setting and inner setting (Appendix 3) ^30^.

***Step 5: Identifying applicability and transferability issues***

This phase will be carried out later as part of the overall research program, through a structured consultation with stakeholders in Canada and internationally.

### Dissemination

A rapid review involves gathering and describing existing literature in supporting evidence-based decision-making and implementation science. The results will be presented at relevant national and international conferences and published in a peer-reviewed journal. They will also be disseminated and shared with professional organizations and learned societies to build the capacity of relevant stakeholders and foster systemic change. In addition, the results will be used to inform further work on the research agenda on this topic, both in Canada and in other countries, and future diabetic foot disease prevention projects aimed at improving knowledge and implementation of foot screening initiatives/programs.

### Patient and Public Involvement

Patients and the public were not involved in the preparation of this rapid review protocol. However, the object of this research is based on the insightful expressed needs of patient partners involved in the development of a diabetic foot screening initiative in primary care in Québec, Canada, a component of our research agenda. This rapid review is conducted to support the knowledge to action framework as a part of a wider research program related to the development of a foot-screening program ^58^.

### Limitations

The omission of non-English, non-French literature may potentially introduce bias into the results. Furthermore, there is a risk that the rapid search may overlook relevant literature due to the potential limitations of this protocol. There is also a risk of missing data in published studies, preventing a complete picture of the implementation components of the foot screening initiative/program. The heterogeneity of the research designs and reported data has the potential to limit secondary analysis and its scope as well as the lack of standardization in diabetic foot screening DF screening and the subjectivity of many screening tests.

## Discussion

IWGDF guidelines recommend that all individuals with diabetes should be assessed, at least once a year, to identify at-risk feet and prevent DFUs and LEAs ^49^. New data has also shown that for individuals at very low risk, once every two years would be sufficient ^59^.

This is not currently the case in Canada ^29^. While many other countries perform better regarding foot screening ^28, 59, 60^, whether through a structured program or routine intervention, the fact remains that there are gaps in the literature demonstrating the added value of investing in a large-scale population-based screening program ^25^. It has been clearly demonstrated that DFU/LEA risk factor screening meets many of the principles of public health to enhance equity. However, better data are needed to assess the true benefits of an organized population-wide diabetes foot screening program before decision and policy-makers invest in nationwide programs ^25^. Thus, it is essential to better support the implementation of foot screening initiatives and programs with the best standards in a knowledge-to-action approach ^58^.

Carrying out a rapid literature review aimed at performing a secondary analysis of recent data on foot screening initiatives/programs, using CFIR, has the potential to raise standards when it comes to the components of implementation science as well as the reporting of articles on the subject. Data will support the team to produce an evidence-based matrix to serve as guidance for implementing foot screening initiatives/programs. The validation phase with stakeholders will ensure pragmatic applicability and knowledge mobilization. Additionally, the insights from this research are expected to significantly impact the development and enhancement of screening programs for diabetic foot complications in primary care.

This knowledge synthesis holds the potential to contribute to the evidence base, providing valuable guidance for healthcare professionals, organizations, decisions and policymakers. Ultimately, the aim is to enhance the overall quality of life for individuals with diabetes, reduce DFU and LEA rates, and lower mortality.

## Declarations

### Ethics

Ethical approval is not required for this research design.

## Data Availability

No data are associated with this article. Open Science Framework: Exploring diabetic foot screening programs with integrated consolidated framework for implementation: Rapid review protocol, 10.17605/OSF.IO/GHYXZ ^61^ This project contains the following underlying data: Appendix 1: Prisma-P Appendix 2: Search Strategy Appendix 3: CFIR References Data are available under the terms of the Creative Commons Attribution 4.0 International license (CC-BY 4.0).
