# Supplementary material for: Exploring diabetic foot screening programs with integrated consolidated framework for implementation: Rapid review protocol
Source: HRB Open Res. 2025 Apr 4;8:49. [Version 1] doi: 10.12688/hrbopenres.14119.1 (PMC12314480; doi:10.12688/hrbopenres.14119.1)
Supplement: Peer review history

# Reviewer report: Reviewer response for version 1

**DOI:** 10.21956/hrbopenres.15517.r47730

**Published:** 2025-08-13

**Authors:** Candra Kusuma Negara

**Affiliations:** 1 Universitas Lambung Mangkurat Banjarmasin, Kota Banjarmasin, Indonesia

To produce a high-quality scientific article, it is essential to begin with a clearly defined research question and explicitly stated objectives, ideally structured using frameworks such as PICO to ensure clarity and focus. The importance of the study should be justified through a strong background section that highlights current gaps in knowledge and is supported by recent, authoritative references. Selecting an appropriate study design that aligns with the research question—such as a rapid review for time-sensitive topics—contributes to methodological rigor. Adherence to standardized reporting guidelines, such as PRISMA-P or those from the Cochrane Rapid Review Methods Group, enhances transparency and reproducibility. Utilizing established theoretical frameworks like the Consolidated Framework for Implementation Research (CFIR) or the ERIC implementation strategies tool ensures the study is grounded in evidence-based methodology. A thorough and transparent description of methods, including eligibility criteria, data sources, search strategies, and quality appraisal tools, is critical. Additionally, considerations of equity, context-specific relevance, and multidisciplinary collaboration enrich the study's value and applicability. Ethical transparency, clear declaration of funding and competing interests, and systematic referencing are further necessary components. Overall, clear language, logical structure, and effective use of visual aids such as tables and figures contribute to making the article accessible and impactful for an international academic audience.

Is the study design appropriate for the research question?

Yes

Is the rationale for, and objectives of, the study clearly described?

Yes

Are sufficient details of the methods provided to allow replication by others?

Partly

Are the datasets clearly presented in a useable and accessible format?

Yes

Reviewer Expertise:

Medical, surgical, diabetes, ULcer, glycemic

I confirm that I have read this submission and believe that I have an appropriate level of expertise to confirm that it is of an acceptable scientific standard.

# Reviewer report: Reviewer response for version 1

**DOI:** 10.21956/hrbopenres.15517.r47726

**Published:** 2025-07-31

**Authors:** Carrinna Aviaja Hansen

**Affiliations:** 1 Orthopaedic Surgery, Zealand University Hospital, Koege, Denmark; 2 Dept. of Regional Health Research, University of Southern Denmark, Odense, Denmark

The manuscript presents a research protocol for an important study, outlining a rapid review that explores diabetic foot screening programs. The review focuses on preventing diabetic foot ulcers (DFU) and lower extremity amputation (LEA) in individuals at risk of these conditions through the improved implementation of diabetic foot screening programs, particularly within primary care.

 The review will contribute to identifying the components of barriers and enablers to the implementation of screening programs, as well as the strategies needed to support their implementation.

 The study rationale is that DFUs are a major complication of diabetes, leading to significant morbidity, including LEA, reduced quality of life, and increased mortality. General, various, and inconsistent care approaches contribute to health inequities. Structured and preventive actions are needed, of which foot screening may be essential. The Consolidated Framework for Implementation Research (CFIR) is well-known for its ability to assess implementation factors and contexts, making it suitable for identifying barriers and facilitators to implementing foot screening programs.

 The Objectives

1. To define the components of diabetic foot screening initiatives using the CFIR.
2. To describe barriers and facilitators to implementing foot screening programs.
3. To describe implementation strategies to support foot screening initiatives. 

The methodology is a Rapid review rather than a systematic review, chosen due to time and resource constraints. The focus will be on synthesising existing evidence, including various qualitative and quantitative designs, but excluding abstracts, guidelines, protocols, expert opinions, editorials, and grey literature. A combination of database searches, including Medline, CINAHL and EMBASE, to ensure the coverage of standard sources will be employed. 

 The PICO defines the eligibility criteria (inclusion and exclusion criteria) and is based on predefined criteria related to diabetic foot screening, CFIR framework application, and relevant study populations (at risk of DFU/LEA).

 The time frame of the published literature will be from the inception of the database to the publication date. Primary studies will be considered after September 2023, which is the search date of Staniszewska et al. 2024, the most recent review on this topic.

 All studies published in French or English will be considered.

 The data extraction will comprise information on patient demographics, setting, screening frequency, approach, and implementation strategies. 

 The data will be synthesised by a narrative synthesis approach drawing on the CFIR domains supplemented by equity, and focusing on implementation determinants, including barriers, enablers/facilitators, and implementation strategies. Thus, reporting expected outcomes regarding implementation and Intervention/System Outcomes.

 This rapid review is of significance since it will provide a valuable contribution and new knowledge supporting efforts focused on preventing DFUs and LEAs among individuals with diabetes and those at risk. To develop targeted and effective/practical strategies to reduce the burden of DFUs and LEAs and improve foot health outcomes for individuals with diabetes, insight into the barriers and enablers of diabetic foot screening programs is essential.

Is the study design appropriate for the research question?

Yes

Is the rationale for, and objectives of, the study clearly described?

Yes

Are sufficient details of the methods provided to allow replication by others?

Yes

Are the datasets clearly presented in a useable and accessible format?

Not applicable

Reviewer Expertise:

Diabetes-related foot ulcers, type-2 diabetes, prevention, health promotion, involvement, multidiciplinary, qualitative and quantitative, socioeconomic

I confirm that I have read this submission and believe that I have an appropriate level of expertise to confirm that it is of an acceptable scientific standard.

## References

[1] : Exploring diabetic foot screening programs with integrated consolidated framework for implementation: Rapid review protocol. *HRB Open Research* .2025;8: 10.12688/hrbopenres.14119.1 10.12688/hrbopenres.14119.1 40756815 PMC12314480

# Reviewer report: Reviewer response for version 1

**DOI:** 10.21956/hrbopenres.15517.r46716

**Published:** 2025-06-20

**Authors:** Loretta Vileikyte

**Affiliations:** 1 Faculty of Health & Medicine, Lancaster University, Lancaster, England, UK

This is an important study, as it aims to address the gap between the effective health services research and its translation into meaningful patient care outcomes. The authors intend to utilise a commonly used implementation science framework, the Consolidated Framework for Implementation Research (CFIR), to analyse foot screening programs for individuals at risk of DFUs and LEAs, to define their key components and implementation determinants, identify barriers and facilitators, and to describe effective implementation strategies in primary care in Canada.

 The study methodology is sound and includes all the necessary components,- a rapid review, the Preferred Reporting Items for Systematic Reviews and Meta-Analyses Protocol guidelines, the PICO framework. Study quality will be appraised using the Mixed Methods Appraisal Tool and data will be synthesized guided by the CFIR. Data synthesis will focus on implementation determinants, including barriers, facilitators, and implementation strategies.

 I ntroduction

 Please expend on the foot screening situation in Canada, see your ref 29: *“information on foot screening practices among Canadians with diabetes remains sparse. A scoping review identiﬁed the limited frequency and uncertain quality of diabetic foot screening across Canada with only 53% receiving a foot examination by a health-care provider at least once in the past year.” *Please compare with/reference foot screening situation on other HICs.

 Please replace the term “disorganized” with perhaps “inadequate” or “not implemented”?

 (line 8; ref 11): Studies also show that individuals with diabetes **foot complications** fear LEAs more than death, as compared to those without diabetes **foot complications.**

Objectives/Methods

 Please clarify whether the focus is on primary and/or secondary DFU prevention (based on your exclusion criteria, it would appear - on 1 ^st^ DFU prevention- if so, please provide the rationale for focusing on primary prevention)

 PICO Intervention (I) description should be revised as follows: A foot screening program is defined as structured and integrated intervention(s) that can use any of the risk stratification tools and the assessment of vascular (e.g., arterial blood supply, with the use of ankle-brachial pressure index or structured clinical examination of peripheral pulses), neurological (e.g., loss of protective sensation; callus formation and foot deformities, as a result of diabetic neuropathy), as well as evaluation of the appropriateness of footwear, as recommended by the IWGDF ^49^ , for individuals with diabetes. “dermatology” use is not appropriate here.

 Step 2: Search for research evidence. Information sources. Could you please elaborate as to what search terms you intend to use?

Is the study design appropriate for the research question?

Yes

Is the rationale for, and objectives of, the study clearly described?

Yes

Are sufficient details of the methods provided to allow replication by others?

Yes

Are the datasets clearly presented in a useable and accessible format?

Not applicable

Reviewer Expertise:

patient psychological adaptation to neuropathy/foot complications

I confirm that I have read this submission and believe that I have an appropriate level of expertise to confirm that it is of an acceptable scientific standard, however I have significant reservations, as outlined above.

# Reviewer report: Reviewer response for version 1

**DOI:** 10.21956/hrbopenres.15517.r46698

**Published:** 2025-04-28

**Authors:** Daryl Lawson

**Affiliations:** 1 University of Nevada, Reno, USA

**Is the rationale for, and the objectives of, the study clearly described?**

**Supporting Page:** Page 3–4.

**Details:** The rationale is clearly described in the “Introduction” section, which explains the burden of diabetic foot ulcers (DFUs), the need for structured screening programs, and the lack of consensus on implementation in Canada. The objectives are listed on Page 4 under “Objectives” and include defining screening components, describing barriers/facilitators, and identifying implementation strategies using CFIR.

**Is the study design appropriate for the research question?**

**Supporting Page:** Page 4–5.

**Details:** The authors chose a rapid review methodology adapted from the Canadian Dobbins method. Given the aim of synthesizing implementation factors for diabetic foot screening programs, this design is suitable for gathering and analyzing literature efficiently to inform future implementation.

**Are sufficient details of the methods provided to allow replication by others?**

**Supporting Page:** Page 5–6.

**Details:** A detailed methodology is provided, including the use of the PICO framework, database selection (MEDLINE, CINAHL, EMBASE), inclusion and exclusion criteria, and quality appraisal tools (Mixed Methods Appraisal Tool and CFIR). A registration with the Open Science Framework (osf.io/ghyxz) is also referenced, supporting transparency and reproducibility.

**Are the datasets clearly presented in a useable and accessible format?**

**Supporting Page:** N/A (protocol only).

**Details:** Since this is a study protocol, data collection and synthesis are forthcoming. The paper outlines how data will be extracted and analyzed using CFIR, but no actual datasets are yet presented.

Is the study design appropriate for the research question?

Yes

Is the rationale for, and objectives of, the study clearly described?

Yes

Are sufficient details of the methods provided to allow replication by others?

Yes

Are the datasets clearly presented in a useable and accessible format?

Not applicable

Reviewer Expertise:

My research area/content is people with diabetes and DFU. I also research orthopedics, especially in the area of musculoskeletal ultrasound imaging. The link below provides my publications and other scholarly work mentioned above: //daryllawson.academia.edu

I confirm that I have read this submission and believe that I have an appropriate level of expertise to confirm that it is of an acceptable scientific standard.
